# The flow, thermal and mass properties of Soret-Dufour model of magnetized Maxwell nanofluid flow over a shrinkage inclined surface

**DOI:** 10.1371/journal.pone.0267148

**Published:** 2022-04-29

**Authors:** Shahanaz Parvin, Siti Suzilliana Putri Mohamed Isa, Fuad S. Al- Duais, Syed M. Hussain, Wasim Jamshed, Rabia Safdar, Mohamed R. Eid

**Affiliations:** 1 Institute for Mathematical Research, Universiti Putra Malaysia, Selangor, Malaysia; 2 Centre of Foundation Studies for Agricultural Science, Universiti Putra Malaysia, Selangor, Malaysia; 3 Mathematics Department, College of Humanities and Science in Aflaj, Prince Sattam Bin Abdulaziz University, AL Aflaj, Saudia Arabia; 4 Administration Department, Administrative Science College Thamar University, Thamar, Yemen; 5 Department of Mathematics, Faculty of Science, Islamic University of Madinah, Madinah, Saudi Arabia; 6 Department of Mathematics, Capital University of Science and Technology, Islamabad, Pakistan; 7 Department of Mathematics, Lahore College for Women University, Lahore, Pakistan; 8 Department of Mathematics, Faculty of Science, New Valley University, Al-Kharga, Al-Wadi Al-Gadid, Egypt; 9 Department of Mathematics, Faculty of Science, Northern Border University, Arar, Saudi Arabia; Stellenbosch University, SOUTH AFRICA

## Abstract

A mathematical model of 2D-double diffusive layer flow model of boundary in MHD Maxwell fluid created by a sloping slope surface is constructed in this paper. The numerical findings of non-Newtonian fluid are important to the chemical processing industry, mining industry, plastics processing industry, as well as lubrication and biomedical flows. The diversity of regulatory parameters like buoyancy rate, magnetic field, mixed convection, absorption, Brownian motion, thermophoretic diffusion, Deborah number, Lewis number, Prandtl number, Soret number, as well as Dufour number contributes significant impact on the current model. The steps of research methodology are as followed: a) conversion from a separate matrix (PDE) to standard divisive calculations (ODEs), b) Final ODEs are solved in bvp4c program, which developed in MATLAB software, c) The stability analysis part also being developed in bvp4c program, to select the most effective solution in the real liquid state. Lastly, the numerical findings are built on a system of tables and diagrams. As a result, the profiles of velocity, temperature, and concentration are depicted due to the regulatory parameters, as mentioned above. In addition, the characteristics of the local Nusselt, coefficient of skin-friction as well as Sherwood numbers on the Maxwell fluid are described in detail.

## 1. Introduction

Heat transfer is a heat engineering activity including manufacture, applications, transformation, as well as the transfer of heat energy between portable systems. Heat transmission is divided into many types, like thermal conduction, convection, radiation, and energy transfer through phase changes [1]. Heat transfer was obtained by advection mass transport of chemical compounds considered by Engineers, which can be either cold or hot. Although these methods have different features, they usually occur simultaneously in the same system. Heat exchange occurs when a large amount of fluid (gas or liquid) flows to its liquid temperature [2]. Heat transfer is one of the maximum essential commercial processes. at some stage in the commercial quarter, warmness should be brought, subtracted, or extracted from the distribution of one process to every other. In theory, the heat dissipated by a hot liquid is not the same as the heat dissipated by a cold fluid due to the loss of natural heat [3]. The continuous enhancement in surrounding fluid causes the increasing rate of heat transfer, and this condition occurs in chemical engineering procedures [4]. In addition, the polymer extrusion method entails cooling the melted liquid expanded in the cooling gadget is related to the application of metallurgical techniques in chemical engineering procedures [5]. Other polymer liquids such as Polyethylene oxide, a polyisobutylene solution in cetane, which has better electric properties, are endorsed as the flow can be managed by outside magnetic factors [6]. Weight transfer is the total motion of weight from one place; generally, that means a movement, component, component, or component, to every other. Bulk transfer occurs in many methods, which include absorption, distillation, drying, evaporation, membrane filtration, and precipitation [7].

Nanofluids are solid-liquid composites made of solid particles of nanometer size, fibers, rods, or tubes hanging from different basic liquids [8]. This fluid type provides promising technological options to improve heat transfer due to its many advantages without exceptional high-temperature fluctuations. Nanofluids represent stepped forward balance compared to conventional liquids supplemented with solid particles of micrometer- or millimeter size due to the size effect and Brownian movement of nanoparticles in liquids. Nanofluids can glide easily into a microchannel with extremely-first-rate nanoparticles without clogging, and the smaller device can be chosen for green heat transfer efficiency. Many researchers have extensively examined solid nanoparticles that disperse nanofluids like metals, carbon nanotubes, and nonmetal nanoparticles. In recent years, a new type of nanofluid dispersed by nanodroplets has been introduced [9]. These nanoemulsion liquids are long-lasting and can be generated easily in bulk. Although the capability of nanodroplets in improving the thermal conductivity of nanofluids can be questioned, improvement in nanoemulsion fluids may open an index of analyses about thermal fluid [10]. The heat transfer efficiency is defined by the convective heat transfer coefficient, which can be stated as many thermo-physical features of nanofluid. This coefficient is critical for thermal conductivity, direct heat, viscosity, and density. Ma [11] introduced a new system consisting of nanoparticles together with liquid metal. From improvement in nanofluid systems, a defining system of nanofluids requires to be changed. Currently, investigations into various nanofluid systems are in the testing phase, and the engineering use of a special nanofluid device is not often suggested. The nanoparticles commonly used to prepare nanofluids reported in the literature are (1) metal particles (Cu, Al, Fe, Au, and Ag); (2) non-metallic particles (Al_2_O_3_, CuO, Fe3O4, TiO2, and SiC); (3) carbon and tuberculosis; and (4) nanodroplet. The top selected basic fluids are oil, ethylene glycol, acetone, water, and decyne [12]. Theory regarding Prandtl boundary layer has proved very useful and has given great impetus to the study of liquid machinery since the turn of the century. One of the most important uses of borderline theory is the calculation of dragging bodies on the flow, e.g., pulling a flat plate in a zero position, a tug-of-war, airfoil, aircraft body, or turbine blade [13]. The current analysis is conducted under the effect of absorption along with injection about heat transfer together with pseudo-plastic non-Newtonian nanofluid flow passing through the permeable sheet. By injection and non-injection plate, a better performance about heat transfer is obtained from non-Newtonian nanofluid than Newtonian nanofluid. Meanwhile, when a type of nanoparticles is changed, there may be a widespread effect on the manner of heat transfer while absorption [14].

Problem construction is done with the Buongiorno model, which combines the features of the two speed slides: Brownian diffusion and thermophoresis. Impacts regarding Brownian movement along with thermophoresis on heat and mass transfer as well as flow from a flat plate with a fixed temperature flux are analyzed by Buongiorno [15]. Test links for active density, thermal conductivity, and viscosity are included in control statistics. Therefore, Buongiorno [15] proposed another model for the convective heat transfer process that is unusual for nanofluids and helps eliminate defects of homogeneous and dispersion models. According to the Buongiorno model, nanoparticles cannot affect dispersion. With these findings as a basis, a two-part non-homogeneous mathematical model of variable transfer was introduced in nanofluids. The above-mentioned model was utilized by Kuznetsov and Nield [16] to examine nanoparticles’ effect on convection boundary-natural surfaces across a straight sheet. Tzou [17] employed it to analyze Bernard’s convection of nanofluids. Hwang et al. [18] utilized it for forced laminar flow convection analysis. A thorough study of the convective transfer of nanofluids was done by Nield and Kuznetsov [19, 20] for boundary-layer flow in a porous medium. The reader may read [4, 21–25] for more interest.

A point-by-step analysis of the 2-D flow design of Maxwell nanoparticles is considered. The formation of nanoparticles is tested in the existence of microorganisms that ensure the stability of nanomaterials [26]. Liquids called non-Newtonian fluids are frequently used in everyday lives in biotechnologies, astrophysics, geophysics, industries, as well as engineering development. So, researchers are intrigued by the analysis of non-Newtonian fluids as they may be analyzed as a different fluid, dosage, and type. The fluid of the combined type is one in which true Cauchy pressure is obtained by a critical analysis of the normal gradient background of aging. A wide variety of fluids is what your pressure is attained by velocity and your many outgoing peaks. When the motion stops with different amounts of pressure fluid, the pressure decreases into an uncertain circular pressure. Compared to a different type of fluid, where pressure is evenly divided as a speed gradient function and its peak output, the fluid type models have a stated relationship between pressure and its peak flow. Maxwell fluid is a viscoelastic fluid that is related to a non-Newton liquid phase. According to experiments, scale-type models are very realistic, requiring both memory and extensor impacts [27]. Hence, Maxwell’s model, which is a subset of standard models, was added to the analysis. Maxwell gave the idea of Maxwell fluid [28]. Most fluids act as Maxwell fluid in the body, like glycerin, engine oil (EO), and other plyometric substances. Various research has been done on Maxwell’s fluid. The Maxwell nanofluid flow over a plate was inspected by Aman et al. [29]. The Laplace modification approach is used to gain an accurate result for the different calculations that are part of initial and boundary constraints. Arif et al. [30] examined Maxwell nanofluid flow to examine the application of their effects to EO on ramped wall constraints. Khalid et al. [31] acquired the effects of ferrofluid on an access station near a vibrating plate with temperatures on the Ramped as well as an isothermal wall. Zhao et al. [32] presented the flow effects of Maxwell nanofluid on heat and mass transfer behavior by Dufour impacts using the Caputo fractional model. Features regarding wave motion happening on Maxwell nanofluid rheometer were examined by Huilgol [33]. Jamil [34] interpreted the flow of Maxwell nanofluid and vibration plate, the result of a shear-compressed shear pressure using a smart Laplace transformer to obtain a closed type of solution. The flexible flow of Maxwell’s non-volatile nanofluid upon an infinitely stable plate with flexible properties was explored by Anwar et al. [35]. Na et al. [36] examined the free flow of Maxwell nanofluid between straight sheets and thermal radiation impact. Raza and Asad [37] considered a flat vertical plate to find solutions to Maxwell fluid flow. It was noted that increase in liquid velocity relative to Grashof (*Gr*) number and Maxwell parameter [38]. Sui et al. [39] found numerical solutions to calculate Maxwell nanofluid over a stretchable sheet that affects heat and mass transfer using a technique of homotopy analysis. Wang and Tan [40] discussed Maxwell nanofluid flow past a tunnel that can penetrate Soret effect. Finally, various mathematical models have been developed to use the heat of nanofluids [22] among them we find effective thermal conductivities from Maxwell’s theory extensions [41–43].

Magnetohydrodynamics (MHD; and magneto-fluid dynamics or hydro-magnetics) in the field of magnetism and electrical properties in the fluid. This magneto-fluid is plasma, fluid metals, electrolytes, and saltwater. MHD statistics are considered along with Rayleigh-Taylor instability, linear and systemic parameters, toroidal instability, high beta tokamaks, nonlinear instability theory, instability resistive, and comparisons between theory and experimentation. MHD measurement characteristics are evaluated, considering energy balance estimates, overvalue, *q* value, Grad-Shafranov equation, an example of bifurcation related to a long cross cylinder, plasma compression between run-down walls, and equilibrium [44]. Since 1960, the principle of MHD actuation developed mainly in the MHD propulsion [45, 46] model reflects the basic goal of MHD micropump, in which Lorentz’s power is generated in *z*−direction. MHD micropump using liquid 1 order / S liquid or aqueous solutions has not been reported yet although micropump MHD containing active mercury fluid was reported in 1993 [47]. Major uses of MHD pumps will be discussed, such as seawater pumping, molten metal, molten salt, and nanofluid pumps. MHD works in astrophysics, including stars, space between planets called interplanetary medium, and possibly even space between stars known as an interstellar medium as well as jets. Formerly, theories describing the formation of the solar and the planets couldn’t explain that the sun has ninety-nine.87% weight, yet only zero.54% of the angular pressure within the solar gadget [48]. Possible use of the MHD flow is that it allows its heat to be converted into electrical energy where the MHD generator is used. The flow of MHD has an extensive function in the form of rotating cylinders in the field of natural sciences such as astrophysics and geophysics. Hayat et al. [49] consider two problems of the Oldroyd-B liquid model over a continuous oscillating plate before conducting heat and electricity when the whole system rotates normally on an oscillatory plate. Darcy’s modified version is used to find the oscillatory flow characteristics of Burger fluid in a hollow area by Hayat et al. [50]. Golinia et al. [51] analyzed various apparent impacts such as fluid flow and magnetic field in the Eyring-Powell fluid and the homogeneous-heterogenous reaction due to the rotating disk and come to an end that the temperature profile had a negative correlation with *Pr* and increased. Of *Nt*. Hayat et al. [52] observed a magnetic field effect on a 2D degree of fluid flow between two parallel discs. Jha and Aina [53] have computerized the reactions of hydromagnetic fluid flow on compressed, viscous fluid and heat-driven in a small vertical horizontal position, developed by irreversible hot plates flowing from side to side. of immediate effect magnet [54].

According to the above-reported articles, the current paper is upgrading the model on double-diffusive convection of Maxwell nanofluid flow, restricted by a stretching surface [55, 56]. The novelty of this paper is substituting the linear function of stretching sheet into exponential function and adding the effect of a magnetic field from [57]. Besides, the additional impact of parameter which is defined as i) the ratio of momentum diffusivity (kinematic viscosity) to thermal diffusivity, and ii) the ratio of thermal diffusivity to mass diffusivity, are considered in the previous model [56]. Since the numerical results in this study are dual, stability analysis is implemented to select the most relevant in an actual fluid situation [56–65, 68–71] for various types of fluid and boundary conditions.

## 2. Methodology

### 2.1 Problem preparation

The problem formulation is under the following restriction:

◾ Two-dimensional Cartesian coordinate model (horizontal and vertical vector).
◾ Incompressible, viscous, and electrically conducting Maxwell fluid
◾ Magnetohydrodynamic model
◾ Heat and mass transfer are occurred simultaneously (Soret-Dufour effects).
◾ Fluid is bounded by the sheet which is shrieked by an exponential function *u*_*w*_(*x*).
◾ The sheet is projected by angle *α* from a fixed *x*−axis [56, 62–64].

The constitutive equation of the Maxwell fluid is

−pI+S=T

$$S+\varepsilon [dS/dt-LS-{SL}^{T}]=\mu A$$ (1)

where *T*, *I*, *L*, *A*, *ε and μ* are declared as Cauchy stress tensor, identity tensor, velocity gradient, first Rivlin–Eriksen tensor, fluid relaxation time and dynamic viscosity of a fluid. Meanwhile,

the derivative of the material is indicated by *dS*/*dt*.

**Fig 1:**
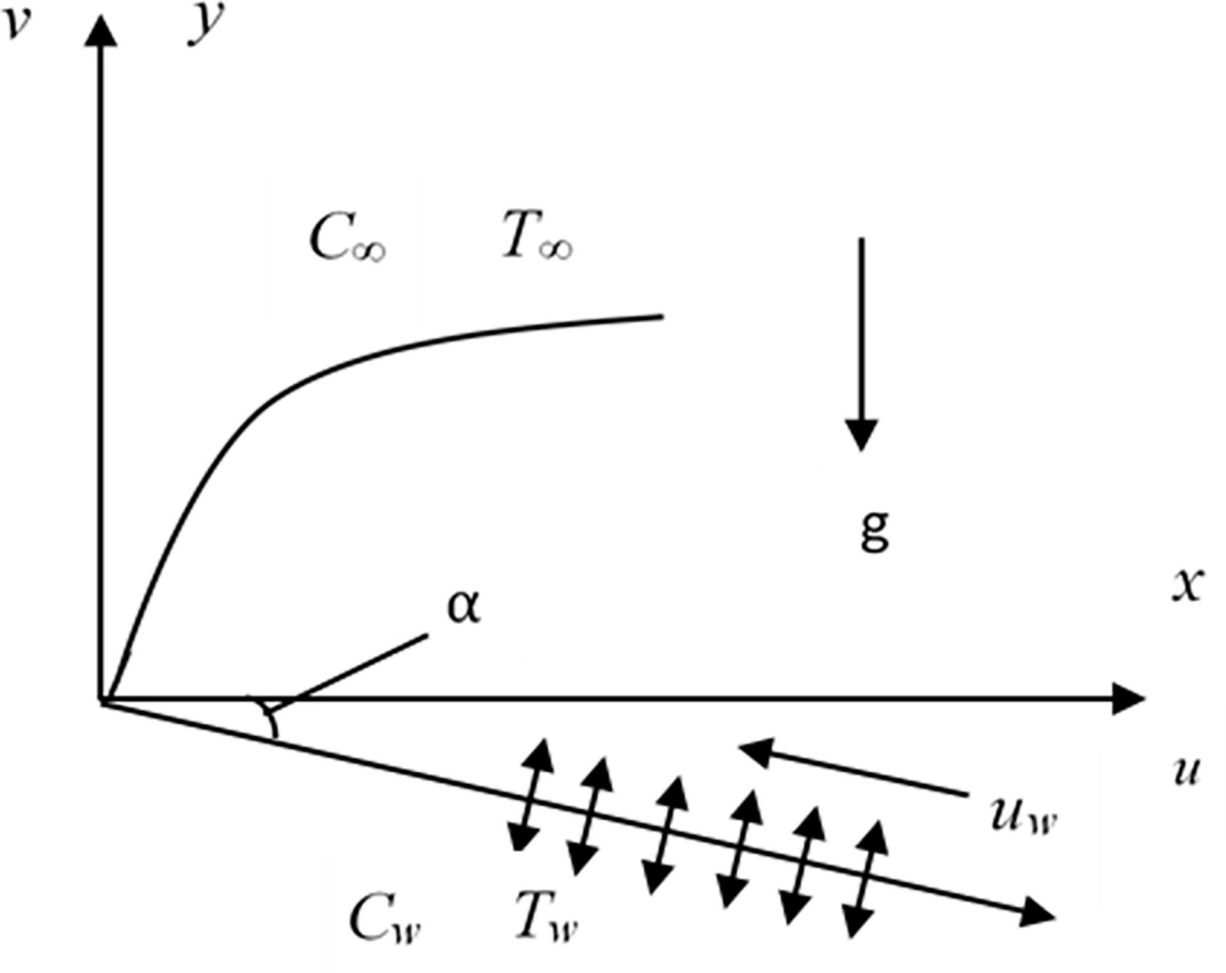
2D model of the fluid flow model.

Fig 1. depicts the representation of the model. Mathematical formulae representing the considered problem can be written as below:

$$u_{x}+v_{y}=0$$ (2)

$$uu_{x}+vu_{y}=\upsilon u_{yy}-\varepsilon (u^{2}u_{xx}+v^{2}u_{yy}+2uvu_{yx})-(\upsilon /K)u+g[\beta_{T}(T-T_{\infty })\cos\;\alpha +\beta_{C}(C-C_{\infty })cos\;\alpha ]-(\sigma {B_{0}}^{2}/\rho )u$$ (3)

$$uT_{x}+vT_{y}=\alpha_{m}T_{yy}+(D_{e}K_{T}/C_{s}C_{p})C_{yy}+\omega [D_{B}C_{y}T_{y}+(D_{T}/T_{\infty }){\left(T_{y}\right)}^{2}]$$ (4)

$$uC_{x}+vC_{y}=(D_{e}K_{T}/T_{m})T_{yy}+D_{B}C_{yy}+(D_{T}/T_{\infty })T_{yy}$$ (5)

where *u* and *v* are velocity components in *x* and *y* directions, *υ* = *μ*/*ρ* is kinematic viscosity, *ρ* is the fluid density, *K* is the permeability constant, *g* is gravitational acceleration, *σ* is representing electrical conductivity, *β*_*T*_ is coefficient of thermal expansion, *β*_*C*_ is the coefficient of concentration expansions, *B*_0_ is uniform strength of the magnetic field, *α*_*m*_ is the thermal diffusivity, *D*_*e*_ is the mass diffusivity, *K*_*T*_ is the thermal diffusion ratio, *C*_*s*_ is the concentration susceptibility, *C*_*p*_ is the specific heat at constant pressure, *ω* is the ratio of effective heat capacity of nanoparticle and base fluid, *D*_*B*_ is the Brownian motion coefficient, *D*_*T*_ is the thermophoretic diffusion coefficient and *T*_*m*_ is the mean fluid temperature. The fluid temperature and concentration are denoted by *T* and *C*, respectively.

The subscript *w* and ∞ denoted the values of *u*, *v*, *T*, and *C* at the wall at and the infinity point, respectively. The partial derivatives acting on *u*, *v*, *T* and *C* concerning *x* and *y* are denoted by the subscripts (*x* and *y*).

The related boundary settings for exponential functions for fluid velocity, temperature, and concentration [58, 64–69] are

$$\begin{array}{l} v=v_{w}(x),u=u_{w}(x)=\lambda U_{0}e^{X},\\ C_{w}(x)=C_{\infty }+C_{0}e^{X},T_{w}(x)=T_{\infty }+T_{0}e^{X}\;at\;y=0,\\ u\to 0,T\to T_{\infty },C\to C_{\infty }\;as\;y\to \infty . \end{array}\}$$ (6)

In addition, a stretching parameter is denoted by *λ*<0, and *X* is presented by *x*/*L* where *L* is the reference length of the shrinking sheet.

Similarity variables will be introduced [68–71]:

$$\begin{array}{l} \theta =(T-T_{\infty })/{(T}_{w}-T_{\infty }),\phi =(C-C_{\infty })/{(C}_{w}-C_{\infty }),\eta =y[\exp\left(X/2\right)]{\left(U_{0}/2L\upsilon \right)}^{1/2},\\ \;u=U_{0}[\exp\left(X\right)]f_{\eta },v=-{\left(\upsilon U_{0}/2L\right)}^{1/2}[\exp\left(X/2\right)][f+\eta f_{\eta }]. \end{array}\}$$ (7)

Where subscript *η* indicates derivative for *η*, wherever *η* is margin layer width. Moreover, *f*, *ϕ* and *θ* in (7)–(13) are functions of *η*.

Implementation of similarity variables (7) into governing equations produce the following ODE

$$f_{\eta \eta \eta }-2{\left(f_{\eta }\right)}^{2}+ff_{\eta \eta }+(\beta )[6ff_{\eta }f_{\eta \eta }-4{\left(f_{\eta \eta }\right)}^{3}-{\left(f\right)}^{2}f_{\eta \eta \eta }]+(\beta )\eta {\left(f_{\eta }\right)}^{2}f_{\eta \eta }+2Ri(\cos\;\alpha )[\theta +N\phi ]+[M-\delta ]f_{\eta }=0,$$ (8)

$$(1/Pr)\theta_{\eta \eta }-\theta f_{\eta }+f\theta_{\eta }+Db\;\phi_{\eta \eta }+Nb\;\phi_{\eta }\theta_{\eta }+Nt\;\theta_{\eta }^{2}=0,$$ (9)

$$\phi_{\eta \eta }+\mathrm{Pr}\;Le[f\phi_{\eta }-\phi f_{\eta }]+[Sr\;\mathrm{Pr}\;Le+(Nt/Nb)]\theta_{\eta \eta }=0$$ (10)

where the controlling parameters occurred in (8)–(10). These equations are subjected to the boundary constraints (BC):

$$\begin{array}{l} f=S,\;f_{\eta }=\lambda ,\varphi =1,\theta =1,\;\mathrm{at}\;\eta =0,\\ f_{\eta }\to 0,\;\phi \to 0,\;\theta \to 0,\;\mathrm{as}\;\eta \to \infty . \end{array}\}$$ (11)

The parameters that occurred in (8)–(11) are listed in Table 1.

**Table 1:** The controlling parameters in ODE and BC.

Controlling Parameters	Equation
Dufour number	$Db=D_{e}K_{T}(C_{w}-C_{\infty })/C_{s}C_{p}(T_{w}-T_{\infty })\upsilon$
Lewis number	*Le* = *α*_*m*_/*Db*
Concentration buoyancy parameter	$N=(C_{w}-C_{\infty })/(T_{w}-T_{\infty })(B_{C}/B_{T})$
Brownian motion parameter	$Nb=\omega D_{B}(C_{w}-C_{\infty })/\upsilon$
Thermophoresis Parameter	$Nt=\omega D_{T}(T_{w}-T_{\infty })/T_{\infty }\upsilon$
Magnetic field parameter	$M=(2\sigma L{B_{0}}^{2}/\rho U_{0})[\exp\left(-X\right)]$
Prandtl number	*Pr* = *υ*/*α*_*m*_
Mixed convection parameter	$Ri=(g\beta_{T}T_{0}L/U_{0}^{2})exp(-3X/2)$ *Ri*<0: opposing flow parameter *Ri*>0: assisting flow parameter
Suction parameter	$S=[v_{w}(x)/\exp\left(X/2\right)]\sqrt{2L/\upsilon U_{0}}>0$
Soret number	$Sr=D_{e}K_{T}(T_{w}-T_{\infty })/T_{m}\upsilon (C_{w}-C_{\infty })$
Deborah number	$\beta =a\lambda_{1}\;\exp\left(X\right)$
Porosity parameter	δ=(υ/aK)exp(−X)

The involved physical parameters are presented by (12) for the case of 2D model of heat and mass transfer with exponential variation at the boundary, with the implementation of Soret-Dufour parameters.

$$C_{f}=(\mu /\rho {U_{0}}^{2})(u_{y}),\;Nu_{x}=(L/(T_{w}-T_{\infty })){\left(-T_{y}\right)}_{y=0},Sh_{x}=(L/(C_{w}-C_{\infty })){\left(-C_{y}\right)}_{y=0}.$$ (12)

where *C*_*f*_ is skin friction coefficient (SFC), *Nu*_*x*_ is Nusselt number and *Sh*_*x*_ is Sherwood’s number.

Substitute (7) into (12) to produce

$$\begin{array}{l} f_{\eta \eta }(0)=C_{f}[\exp\left(-3X/2\right)]{\left(2Re_{x}\right)}^{1/2},-\theta_{\eta }(0)=Nu_{x}[\exp\left(-X/2\right)]{\left(2/Re_{x}\right)}^{1/2},\\ -\phi_{\eta }(0)=Sh_{x}[\exp\left(-X/2\right)]{\left(2/Re_{x}\right)}^{1/2}, \end{array}\}$$ (13)

where Rex=UoL[exp(X/L)]/υ is the Reynolds number.

### 2.2 Stability analysis

Multiple numerical solutions require the specific technique to determine a most stable solution, which is the actual results that have a high possibility to occur in an actual fluid situation and will be able to become a reference to the experimental results [56–66, 68–71]. Therefore, this section will discuss the steps of how to perform this method, namely stability analysis. The lowest positive eigenvalue indicates the stable and reliable numerical solution. Besides, the lowest negative eigenvalues refer to the unstable solution (which is unreliable and rejected). Therefore, the initial step of this method is introducing the unsteady state of Eqs (3)–(5), as below:

$$u_{t}+uu_{x}+vu_{y}=\upsilon u_{yy}-\lambda_{1}(u^{2}u_{xx}+v^{2}u_{yy}+2uvu_{yx})-(\upsilon /K)u+g\left[\begin{array}{c} B_{T}(T-T_{\infty })\;\cos\;\alpha \\ +B_{C}(C-C_{\infty })\;\cos\;\alpha \end{array}\right]-(\sigma {B_{0}}^{2}/\rho )u,$$ (14)

$$T_{t}+uT_{x}+vT_{y}=\alpha_{m}T_{yy}+(D_{e}K_{T}/C_{s}C_{p})C_{yy}+\omega \;\left[D_{B}C_{y}T_{y}+(D_{T}/T_{\infty }){\left(T_{y}\right)}^{2}\right],$$ (15)

$$C_{t}+uC_{x}+vC_{y}=(D_{e}K_{T}/T_{m})T_{yy}+D_{B}C_{yy}+(D_{T}/T_{\infty })T_{yy},$$ (16)

In the stability analysis part, *f*, *θ*, and *ϕ* in Eqs (14)–(16) are the functions in (*η*, *τ*). Therefore, the boundary constraints are

$$\begin{array}{l} f=S,\;f_{\eta }=\lambda ,\varphi =1,\theta =1,\;at\;\eta =0,\\ \;f_{\eta }\to 0,\varphi \to 0,\theta \to 0,\;as\;\eta \to \infty . \end{array}\}$$ (17)

New similarity variants, which involve dimensionless time variant *τ* are given as

$$\begin{array}{l} \theta =(T-T_{\infty })/{(T}_{w}-T_{\infty }),\phi =(C-C_{\infty })/{(C}_{w}-C_{\infty }),\tau =(U_{0}/2L)[\exp\left(X\right)]\;t,\\ \eta =y[\exp\left(X/2\right)]{\left(U_{0}/2L\upsilon \right)}^{1/2},u=U_{0}[\exp\left(X\right)]f_{\eta },\\ v=-(1/2L){\left(2\upsilon LU_{0}\right)}^{1/2}\{[\exp\left(X/2\right)]f_{\eta }+(U_{0}/L)[\exp\left(\frac{3X}{2}\right)]tf_{\tau }+[\exp\left(\frac{X}{2}\right)]f\}. \end{array}\}$$ (18)

When (18) is substituted into (14)–(16), the following is gained:

$$f_{\eta \eta \eta }-f_{\tau \eta }-2{\left(f_{\eta }\right)}^{2}+ff_{\eta \eta }-2\tau (f_{\eta }f_{\tau \eta }-f_{\eta \eta }f_{\tau })+\beta \eta {\left(f_{\eta }\right)}^{2}f_{\eta \eta }+\beta e^{X}(6ff_{\eta }f_{\eta \eta }-f^{2}f_{\eta \eta \eta }-4{\left(f_{\eta }\right)}^{3})+4\beta \tau^{2}(2f_{\eta }f_{\tau }f_{\tau \eta \eta }-f_{\eta \eta \eta }{\left(f_{\tau }\right)}^{2}-{\left(f_{\eta }\right)}^{2}f_{\tau \tau \eta })+4\beta \tau (3f_{\eta }f_{\tau }f_{\eta \eta }-3{\left(f_{\eta }\right)}^{2}f_{\tau \eta }-ff_{\tau }f_{\eta \eta \eta }+ff_{\eta }f_{\tau \eta \eta })+(M-\delta )f_{\eta }+2Ri(\cos\;\alpha )[\theta +N\varphi ]=0$$ (19)

$$(1/Pr)\theta_{\eta \eta }-\theta_{\tau }+f\theta_{\eta }-f_{\eta }\theta +2\tau f_{\tau }\theta_{\eta }-2\tau f_{\eta }\theta_{\tau }+D_{f}\phi_{\eta \eta }+Nb\theta_{\eta }\phi_{\eta }+Nt{\left(\theta_{\eta }\right)}^{2}=0,$$ (20)

$$(1/Sc)\phi_{\eta \eta }+Sr\theta_{\eta \eta }+(Nt/NbPrLe)\theta_{\eta \eta }-\phi_{\tau }+\phi_{\eta }f-\phi f_{\eta }+2\tau f_{\tau }\phi_{\eta }-2\tau \phi_{\tau }f_{\eta }=0.$$ (21)

The equations related to stability analysis are specified as [72]

$$\begin{array}{l} f=f_{0}+e^{-\gamma \tau }F,\\ \theta =\theta_{0}+e^{-\gamma \tau }G,\\ \phi =\phi_{0}+e^{-\gamma \tau }H. \end{array}\}$$ (22)

where *f*_0_, *θ*_0_ and *ϕ*_0_ are the function of *η*, and *F*, *G*, and *H* are the functions in (*η*, *τ*). Besides, *γ* is the lowest eigenvalue while *F*(*η*, *τ*), *G*(*η*, *τ*) and *H*(*η*, *τ*) are minor comparative to *f*_0_(*η*), *θ*_0_(*η*) and *ϕ*_0_(*η*). Next, (22) is substituted into (17) and (19)‒(21) and set *τ* = 0. Consequently, the following equations are obtained:

$${\left(F_{0}\right)}_{\eta \eta \eta }+\gamma {\left(F_{0}\right)}_{\eta }-4{\left(F_{0}\right)}_{\eta }{\left(f_{0}\right)}_{\eta }+F_{0}{\left(f_{0}\right)}_{\eta \eta }+f_{0}{\left(F_{0}\right)}_{\eta \eta }+\beta f_{0}[6{\left(f_{0}\right)}_{\eta }{\left(F_{0}\right)}_{\eta \eta }+6{\left(F_{0}\right)}_{\eta }{\left(f_{0}\right)}_{\eta \eta }-f_{0}{\left(F_{0}\right)}_{\eta \eta \eta }-2F_{0}{\left(f_{0}\right)}_{\eta \eta \eta }]+\beta {\left(f_{0}\right)}_{\eta }[6F_{0}{\left(f_{0}\right)}_{\eta \eta }-12{\left(f_{0}\right)}_{\eta }{\left(F_{0}\right)}_{\eta }+\eta {\left(f_{0}\right)}_{\eta }{\left(F_{0}\right)}_{\eta \eta }+2\eta {\left(F_{0}\right)}_{\eta }{\left(f_{0}\right)}_{\eta \eta }]+[M-\delta ]{\left(F_{0}\right)}_{\eta }+2Ri(\cos\;\alpha )[G_{0}+NH_{0}]=0,$$ (23)

$$(1/Pr){\left(G_{0}\right)}_{\eta \eta }+Db{\left(H_{0}\right)}_{\eta \eta }+f_{0}{\left(G_{0}\right)}_{\eta }{-G_{0}{\left(f_{0}\right)}_{\eta }}_{}{+F_{0}{\left(\theta_{0}\right)}_{\eta }}_{}-{\theta_{0}{\left(F_{0}\right)}_{\eta }+\gamma G_{0}}_{}+Nb[{\left(H_{0}\right)}_{\eta }{\left(\theta_{0}\right)}_{\eta }+{\left(\phi_{0}\right)}_{\eta }{\left(G_{0}\right)}_{\eta }]+2Nt{\left(G_{0}\right)}_{\eta }{\left(\theta_{0}\right)}_{\eta }=0,$$ (24)

$$Sr{\left(G_{0}\right)}_{\eta \eta }+\gamma H_{0}+f_{0}{\left(H_{0}\right)}_{\eta }-H_{0}{\left(f_{0}\right)}_{\eta }-\phi_{0}{\left(F_{0}\right)}_{\eta }+F_{0}{\left(\phi_{0}\right)}_{\eta }+(1/\mathrm{Pr}Le)[{\left(H_{0}\right)}_{\eta \eta }+(Nt/Nb){\left(G_{0}\right)}_{\eta \eta }]=0.$$ (25)

with the boundary condition from (17)

$$\begin{array}{l} {\left(F_{0}\right)}_{\eta }=0,F_{0}=0,G_{0}=0,H_{0}=0\;at\;\eta =0.\\ {\left(F_{0}\right)}_{\eta }\to 0,G_{0}\to 0,H_{0}\to 0\;as\;\eta \to \infty . \end{array}\}$$ (26)

First boundary condition in (26) is satisfied as *η*→∞ [73] and replaced with one higher order at *η* = 0. This BC is equal to 1. Finally, (23)‒(26) with new boundary condition (*F*_0_)_*ηη*_ = 1 is being solved with the help of MATLAB software to obtain the lowest eigenvalues (where the positive values are the most stable solution and it is denoted by the first solution, otherwise it is unstable and it is indicated by the second solution).

### 2.3 Numerical method

This section describes the final ODEs which have been implemented in MATLAB bvp4c code. The coding contains Codes A, B, C, and D. Code A is implemented to find the numerical solutions for any values of independent variables, whereas Code B is the continuation code that calculates the numerical values at the region close to a critical point. Therefore, the governing Eq (8)–(10) with the boundary condition (11) are implemented in the Matlab bvp4c codes (Codes A and B). Finally, the following substitutions for (8)–(11) are introduced:

(1/(1−βy(1)y(1)))(2y(2)y(2)−y(1)y(3)−β(6y(1)y(2)y(3)−4y(2)y(2)y(2))−βy(2)y(2)y(3)+y(2)(M+δ)−2Ri(y(4)+Ny(6))) (27)

((NbPr)/(Nb−PrDb(SrLePrNb+Nt)))(y(2)y(4)−y(1)y(5)−Nby(5)y(7)−Nty(5)y(5)+DbPrLe(y(1)y(7)−y(2)y(6))) (28)

(−Pr/(Nb−PrDb(SrLePrNb+Nt)))(LeNb(y(1)y(7)−y(2)y(6))+(SrLePrNb+Nt)(y(2)y(4)−y(1)y(5)−Nby(5)y(7)−Nty(5)y(5))) (29)

ya(1)=S,ya(2)=λ,ya(4)=1,ya(6)=1,yb(2)=0,yb(4)=0,andyb(6)=0. (30)

On the other hand, codes C and D are developed to find the lowest eigenvalues (positive values for code C, and negative values on D). In this part, (23)‒(26) with new boundary condition (*F*_0_)_*ηη*_ = 1 are transformed:

((1)/(1−βs(1)s(1)))((−s(1)y(3)−s(3)y(1)+4s(2)y(2)−γy(2)−2Ri(y(4)+Ny(6))−6βs(1)(s(2)y(3)+y(2)s(3))+(M+δ)y(2)−βs(2)(6y(1)s(3)−12s(2)y(2)+s(2)y(3)+2y(2)s(3)))+((2βs(1)y(1))/(1−βs(1)s(1)))(2s(2)s(2)−s(1)s(3)−β(6s(1)s(2)s(3)−4s(2)s(2)s(2))−βs(2)s(2)s(3)+(M+δ)s(2)−2Ri(s(4)+Ns(6)))) (31)

((PrNb)/(Nb−DbPr(SrPrNbLe+Nt)))(−DbPrLe(−γy(6)−s(1)y(7)+y(6)s(2)+s(6)y(2)−y(1)s(7))−2Nty(5)s(5)−s(1)y(5)+y(4)s(2)−y(1)s(5)+s(4)y(2)−γy(4)−Nb(y(7)s(5)+s(7)y(5))) (32)

((Pr)/(Nb−DbPr(SrPrLeNb+Nt)))(NbLe(s(6)y(2)−y(1)s(7)−s(1)y(7)+y(6)s(2)−γy(6))+(SrPrLeNb+Nt)(s(1)y(5)−y(4)s(2)+y(1)s(5)−s(4)y(2)+γy(4)+Nb(y(7)s(5)+s(7)y(5))+2Nty(5)s(5))) (33)

ya(1),ya(2),ya(3)−1,ya(4),ya(6),yb(4),yb(6). (34)

Eqs (27)–(34) are then coded into the bvp4c solver. The syntax of the solver, sol = bvp4c(@OdeBVP,@OdeBc,solinit,options) contains the function handle @OdeBVP (for ODEs) and @OdeBC (for boundary conditions). The values of governing parameters (*Le*, *N*, *Nb*, *Nt*, *M*, *Ri*, *S*, *Pr*, *β*, *Db*, *Sr*, *δ*, *α*, and *λ*) will be fixed in bvp4c solver, together with the highest boundary layer thickness. These values are accepted as long as the numerical results are following the boundary conditions.

### 3. Outcomes and conversation

Graphical representation of SFC Cfe−3X/22Rex, local Sherwood number Shxe−X/22/Rex, and local Nusselt number Nuxe−X/22/Rex, is drawn to show the effect of these controlling parameters, such as Lewis *Le*, buoyancy-ratio *N*, Brownian diffusion *Nb*, thermophoretic parameter *Nt*, magnetic field *M*, mixed convective parameter *Ri*, suction *S*, Prandtl number *Pr*, and Deborah number *β*. These parameters also have been drawn on the profiles of velocity *f*_*η*_ temperature *θ* and concentration *ϕ* against fluid thickness *η*, where maximum *η* is 10. All the numerical results are restricted under the following values of fixed controlling parameters except or else stated: *Le* = 1, *N* = 0.5, *Nb* = 0.8, *Nt* = 0.1, *M* = 0.5, *Ri* = −0.027, *S* = 1.5, *Pr* = 1, *β* = 0.0607, *Db* = 0.5, *Sr* = 0.2, *δ* = 1.6485, *α* = 15°, and *λ* = −0.5.

### 3.1 Verification of current numerical method

Accuracy of the MATLAB bvp4c implemented in this paper is verified by comparing the numerical data of local Nusselt number $Nu_{x}e^{-X/2}\sqrt{2/Re_{x}}$, with Ramzan et al. [55] for increasing *S*, *λ*, and *β* when *Nb* = 0.8, *M* = 0.0, *N* = *Ri* = *Pr* = *Le* = 1.0, *Sr* = 0.2, *δ* = 2.0, *Nt* = 0.1, *Db* = 0.1. The magnetic field parameter (*M* value) is considered zero for the numerical calculation since this is an additional parameter in the current research compared to [55]. On the other hand, the linear boundary condition is applied in the previous researcher’s work [55] whereas the exponential boundary condition is used in the present study. Thus, the stretching parameter **λ** is included in the comparison table which is shown in Table 2. therefore, the coolest assessment in Table 2 proves that our bvp4c MATLAB is applicable for the whole findings in this article.

**Table 2:** Local Nusselt number against *S*, *β*, *λ* when *Nb* = 0.8, *M* = 0.0, *N* = *Ri* = *Pr* = *Le* = 1.0, *Sr* = 0.2, *δ* = 2.0, *Nt* = 0.1, *Db* = 0.1.

*S*	*β*	*λ*	−*θ*′(0)	
			[73]	Present
0.0	0.1	0.8815	0.71104	0.71107
0.3		0.8885	0.79696	0.79694
0.9		0.8430	0.99873	0.99871
0.5	0.0	0.8690	0.86690	0.86682
	0.1	0.8890	0.85983	0.85980
	0.2	0.8514	0.85263	0.85262

### 3.2 Selection of reliable numerical solution

The previous researchers have proved that the dual numerical solutions will be occurred with the presence of suction, together with the suitable rate of shrinkage boundary surface [56, 62–66, 68–71] or extended boundary surface [57–61] in the fluid flow model. The most stable one among the dual numerical solutions is selected by using stability analysis with the aid of MATLAB bvp4c program, as it declares uniform change as the first solution. Meanwhile, another solution will be labeled as the second one. First solution is linearly stable and physically realizable, as well as shows satisfying boundary conditions (Eq (11)) without or with the minimal existence of minimum and maximum peak. Table 3 presents the numerical values for stability analysis, where the smallest eigenvalues for many parametric values of *λ* and *Pr* are given. According to the table, the first solution is obtained with positive smallest eigenvalues, while negative for the other one. In Table 3, the range of the controlling parameters must be close to the critical point *λ*_c_ = -0.4495, where this point is the intersection point that connects the regions of both solutions.

**Table 3:** Smallest eigenvalues for several values of *λ* and *Pr*.

*λ*	Pr	First solution	Second solution
-0.45	1.0	0.13542	-0.03160
	1.5	0.38065	-0.41925
	2.0	0.65072	-0.68531
-0.5	1.0	0.13545	-0.04185
	1.5	0.37165	-0.40752
	2.0	0.65450	-0.67533

### 3.3 Velocity outline

**Fig 2:**
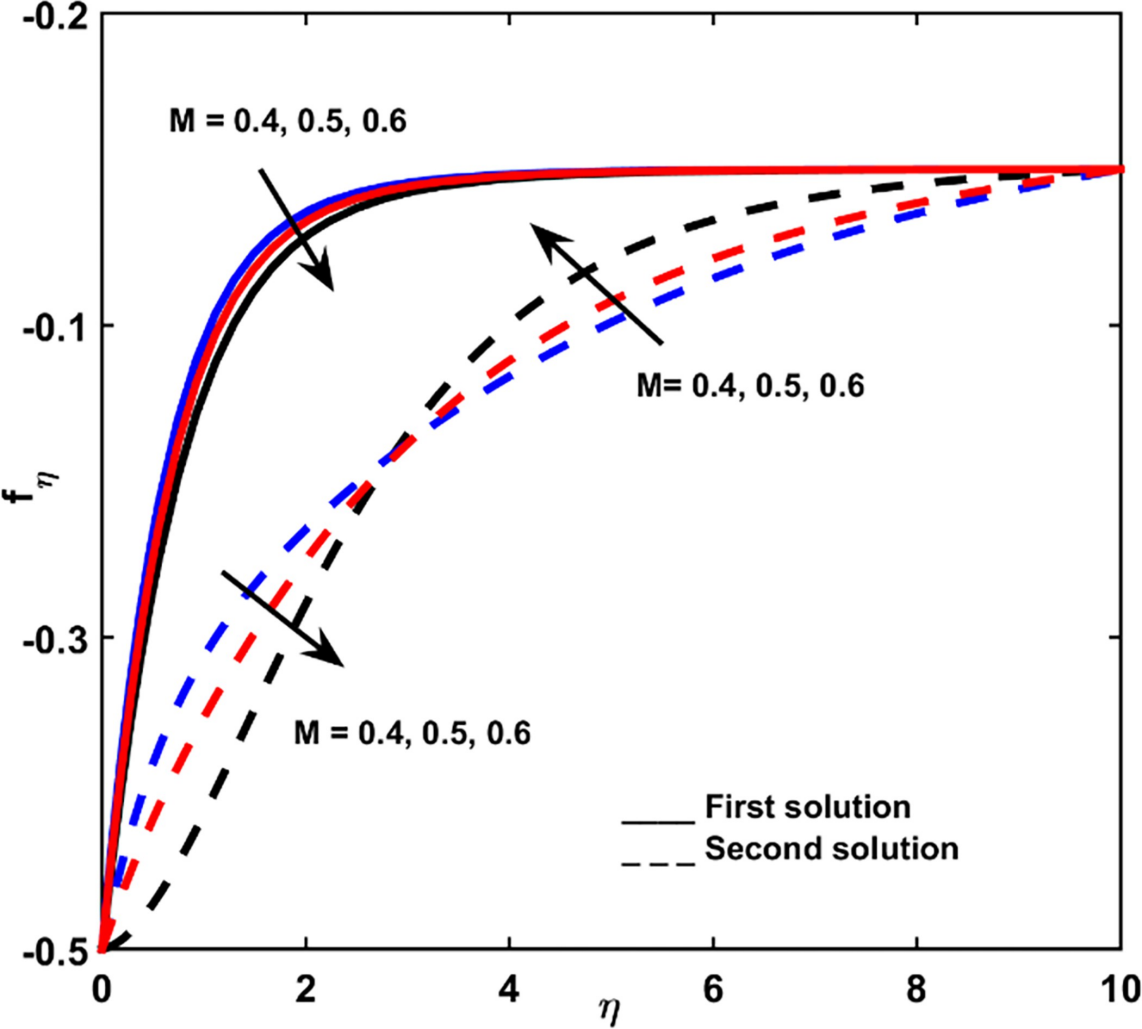
Velocity profile for diverse values of *M*.

**Fig 3:**
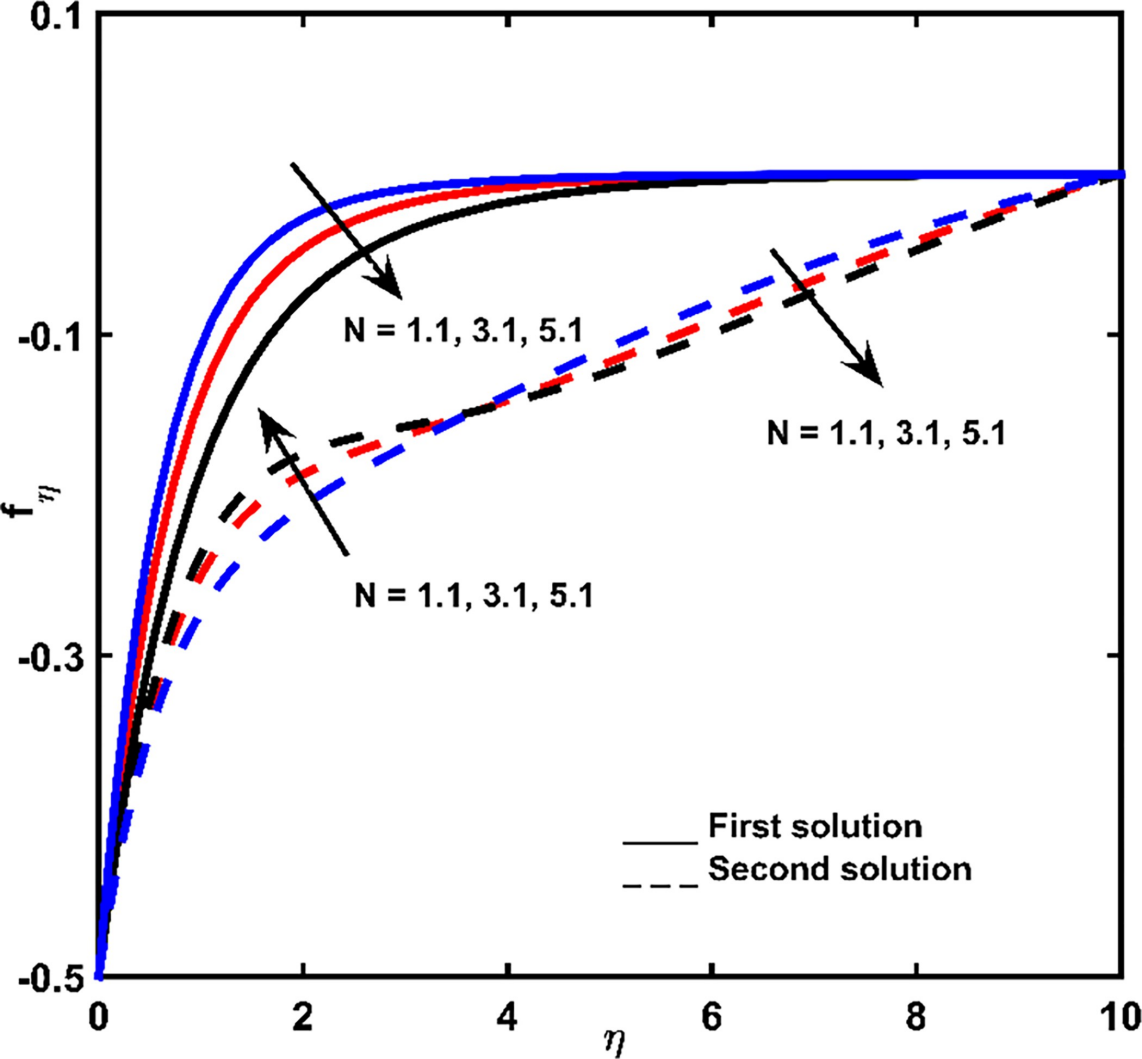
Velocity impact for diverse values of *N*.

Figs 2, 3 is indicating the velocity outlines *f*_*η*_ for changed entities of magnetic force parameter *M* and buoyancy ratio parameter *N*, correspondingly. Notation of negative values regarding *f*_*η*_ is that fluid flow directs in a reverse manner with shrinking vector. As the result, superior negative values of *f*_*η*_ are denoting more velocity in the reverse direction. When fluid is becoming thicker (increasing *η*), for the first result, velocity is gradually decreasing, provided that velocity tends to zero.

As shown in Fig 2, *f*_*η*_ in the first solution increases by increasing *M*. This is due to the force of magnetism acting as an assistant to the fluid flow, for the case when a lessening sheet tempts the fluid flow. The *M* parameter (as exposed in Fig 2) causes the increment on the degree of the velocity of 2^nd^ outcome at the space nearly the lessening sheet, whereas it maximizes when the fluid thickness is higher. While Fig 3 presents the velocity outline for the first outcome growths based on the adding rate in buoyancy ratio.

### 3.4 Temperature profile

**Fig 4:**
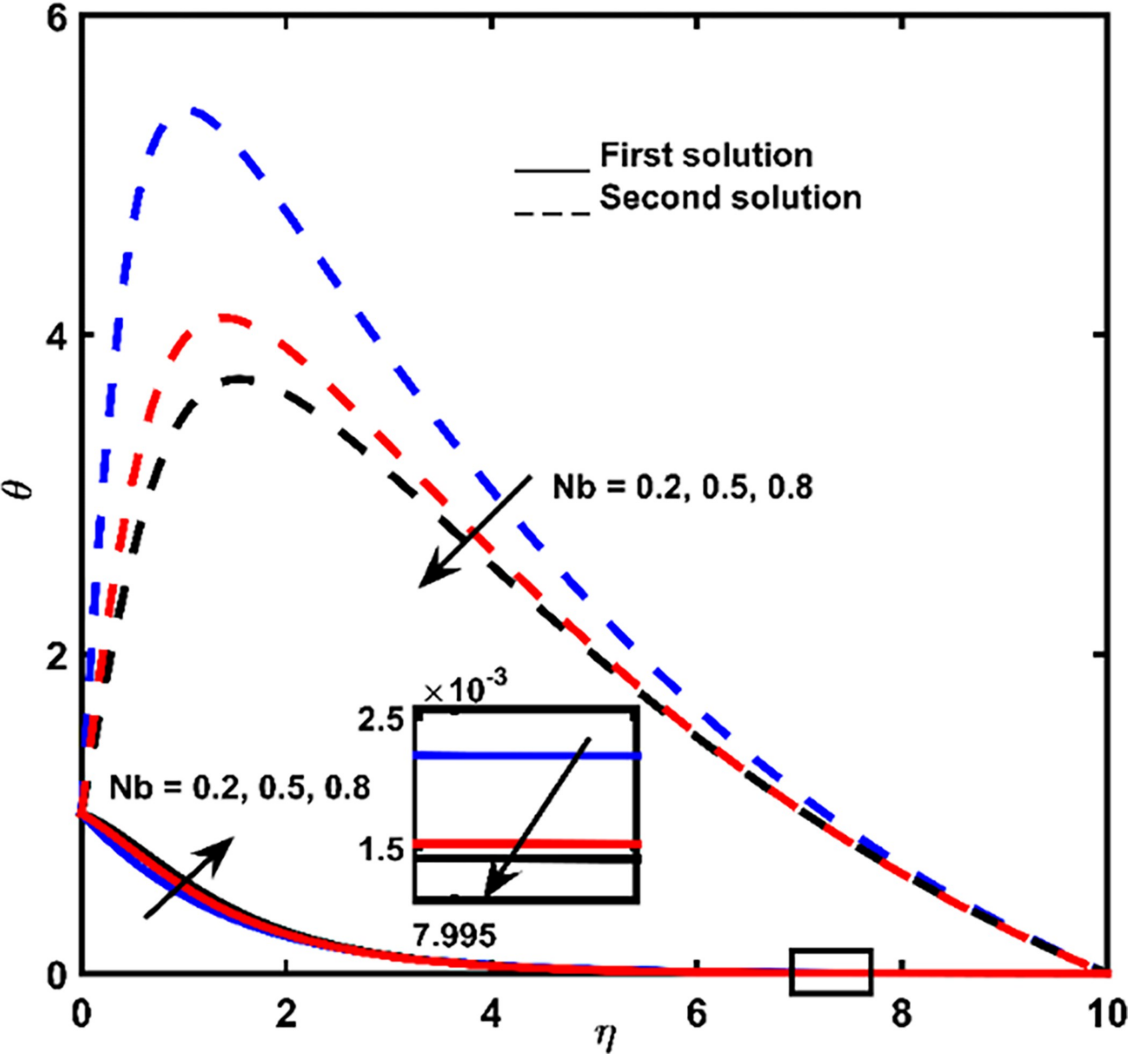
Temperature impact for *Nb*.

**Fig 5:**
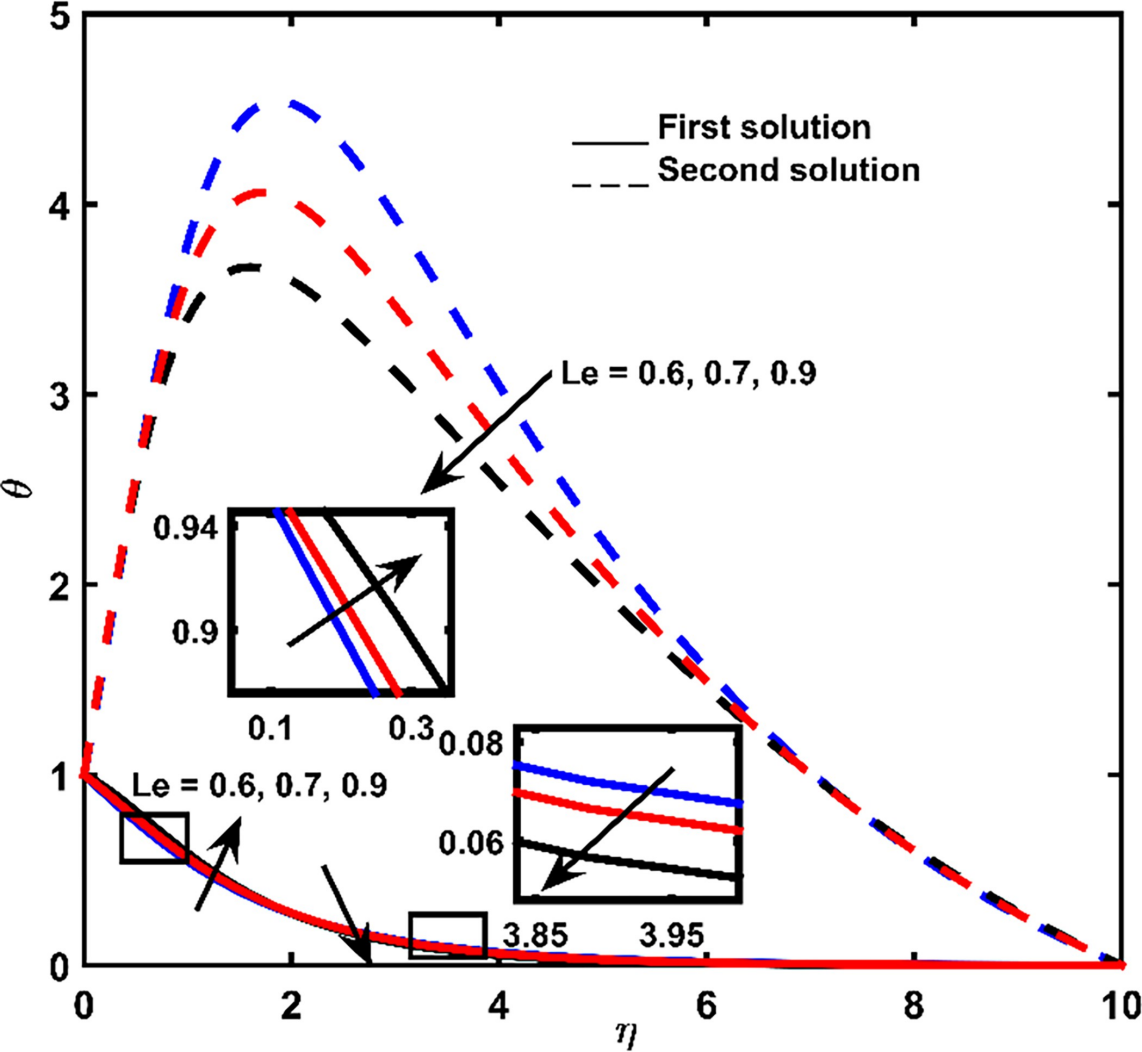
Temperature outline for *Le*.

**Fig 6:**
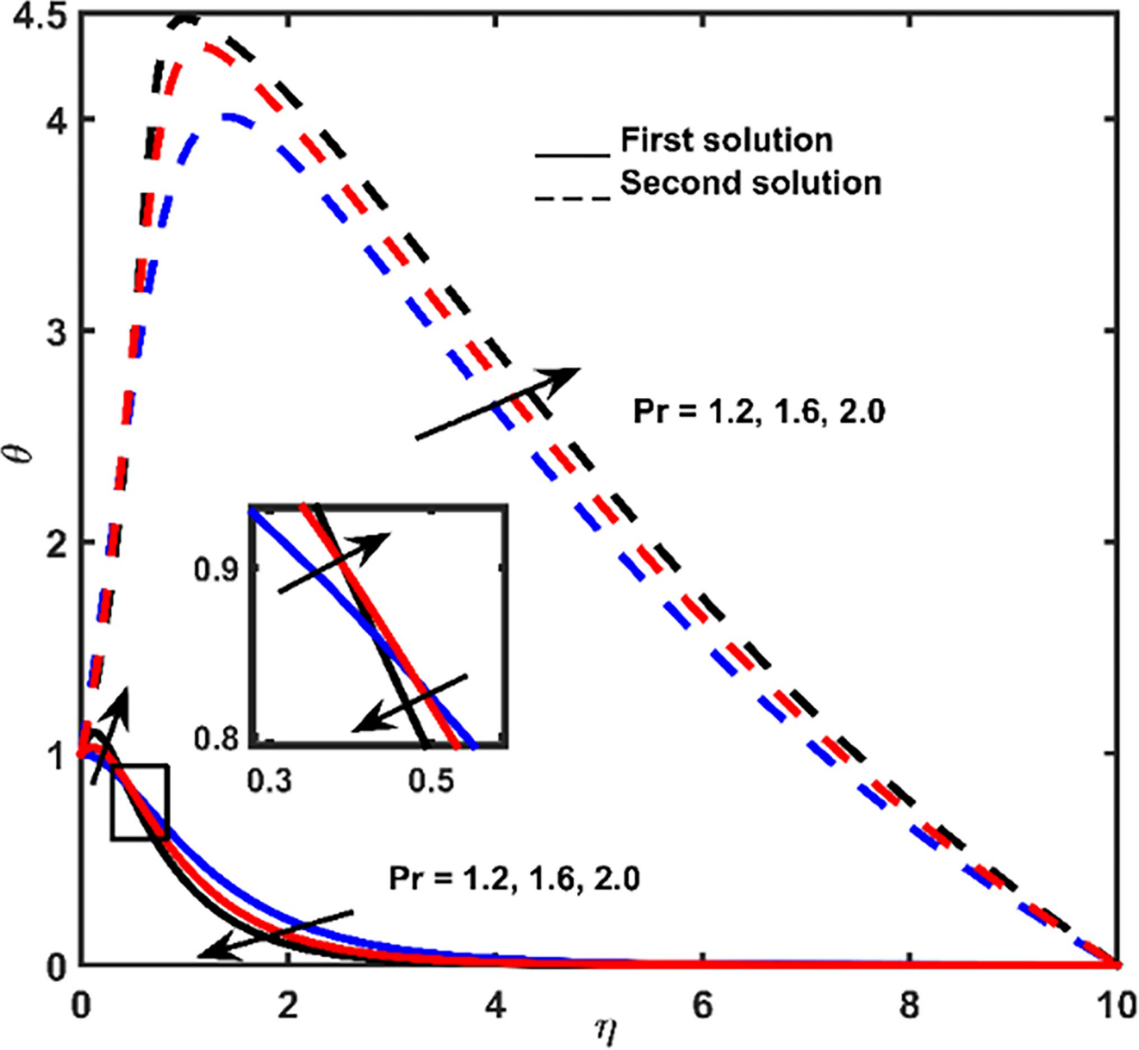
Temperature outline regarding *Pr*.

The effect of Brownian diffusion *Nb*, Prandtl *Pr* number, and Lewis number *Le* on-temperature outlines are depicted in Figs 4–6, respectively. Heat obtained from the first result is gradually vanishing until it approaches zero when it is measured by shrinking surface space. However, heat regarding 2^nd^ results increases until it reaches full heat. Increment in highest point at the closest point. Continuously, heat obtained from the second result tends to zero at a far-off point from the shrinking surface.

Influence about Brownian parameter *Nb* on the temperature of the first solution, which shows in Fig 4 indicates the design of heat difference is nonuniform (increases at a nearby point and consequently decreases at a far point). The inspiring beliefs of Brownian diffusion will grow the non-dimensional heat outlines at small fluid thickness. Brownian motion is defined as a random movement of particles in the liquid. Therefore, the increment of Brownian diffusion will increase the kinetic energy of the particles, which points to the enhancement of the thermal boundary layer width.

The effect of Lewis number *Le* as well as Prandtl number *Pr* on the first solution of *θ* is presented in Figs 5 and 6, respectively. These parameters cause the pattern of heat deviation to be non-regular (increases at adjacent-point and consequently losses at an extreme point). Parameter *Le* is formulated as the thermal ratio of impetus diffusion, whereas parameter *Pr* is the ratio of impetus to thermal diffusion. The significant effect of *Pr* can be observed at large *η* (Fig 6), whereas *Le* at small *η* (Fig 5). Higher *Pr* corresponds to weaker thermal-diffusivity, which decreases the thermal boundary-layer thickness at large *η*. On the other hand, *Le* number due to the thermal diffusivity influences the temperature outline, where higher *Le* induces the increment of temperature boundary-layer thickener at small *η*. In the situation of the second outcome, heat distribution losses with the augmentation of parameter *Le* and increases with the increasing *Pr*.

### 3.5 Concentration profile

**Fig 7:**
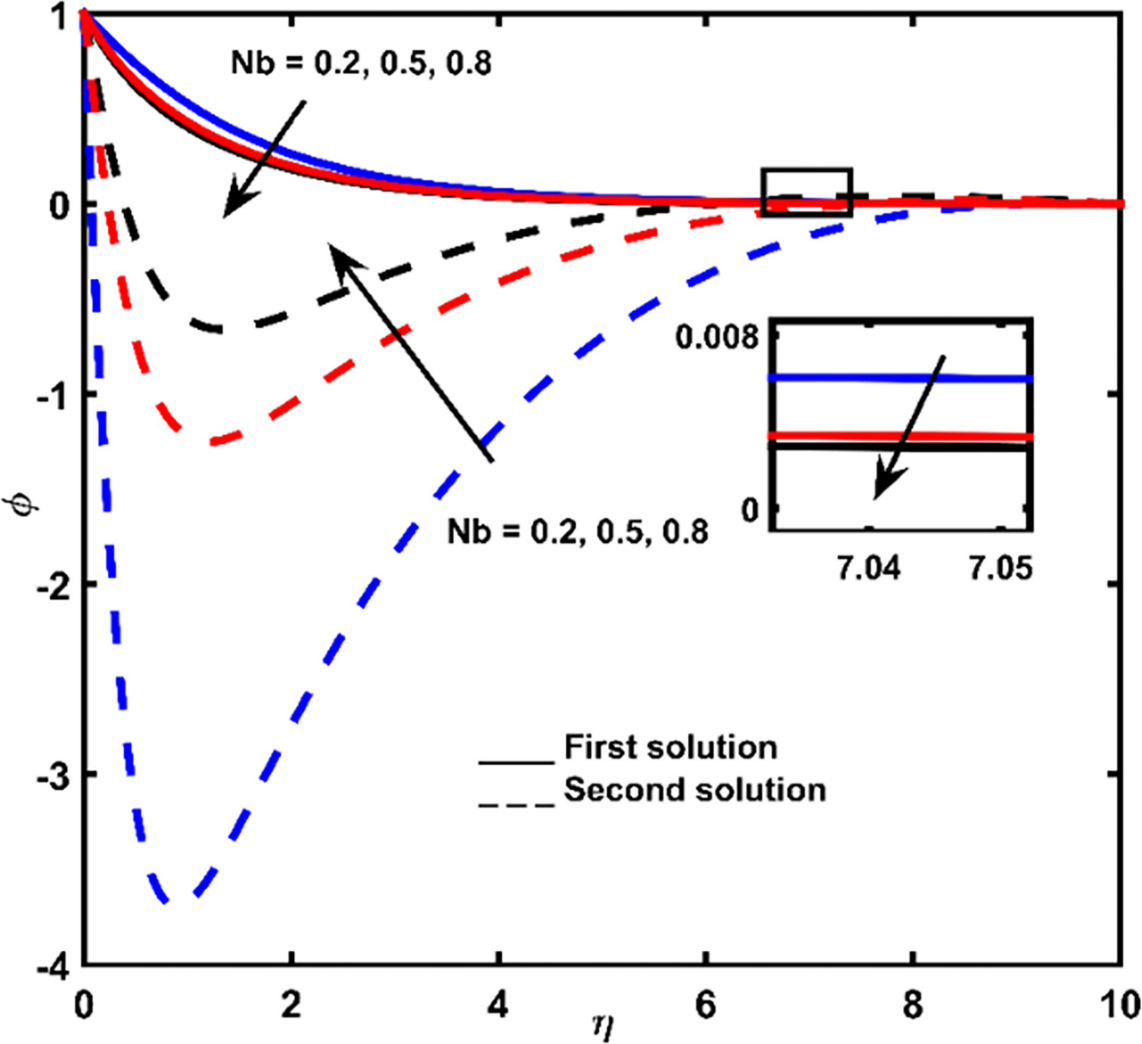
Concentration impact for diverse values of *Nb*.

**Fig 8:**
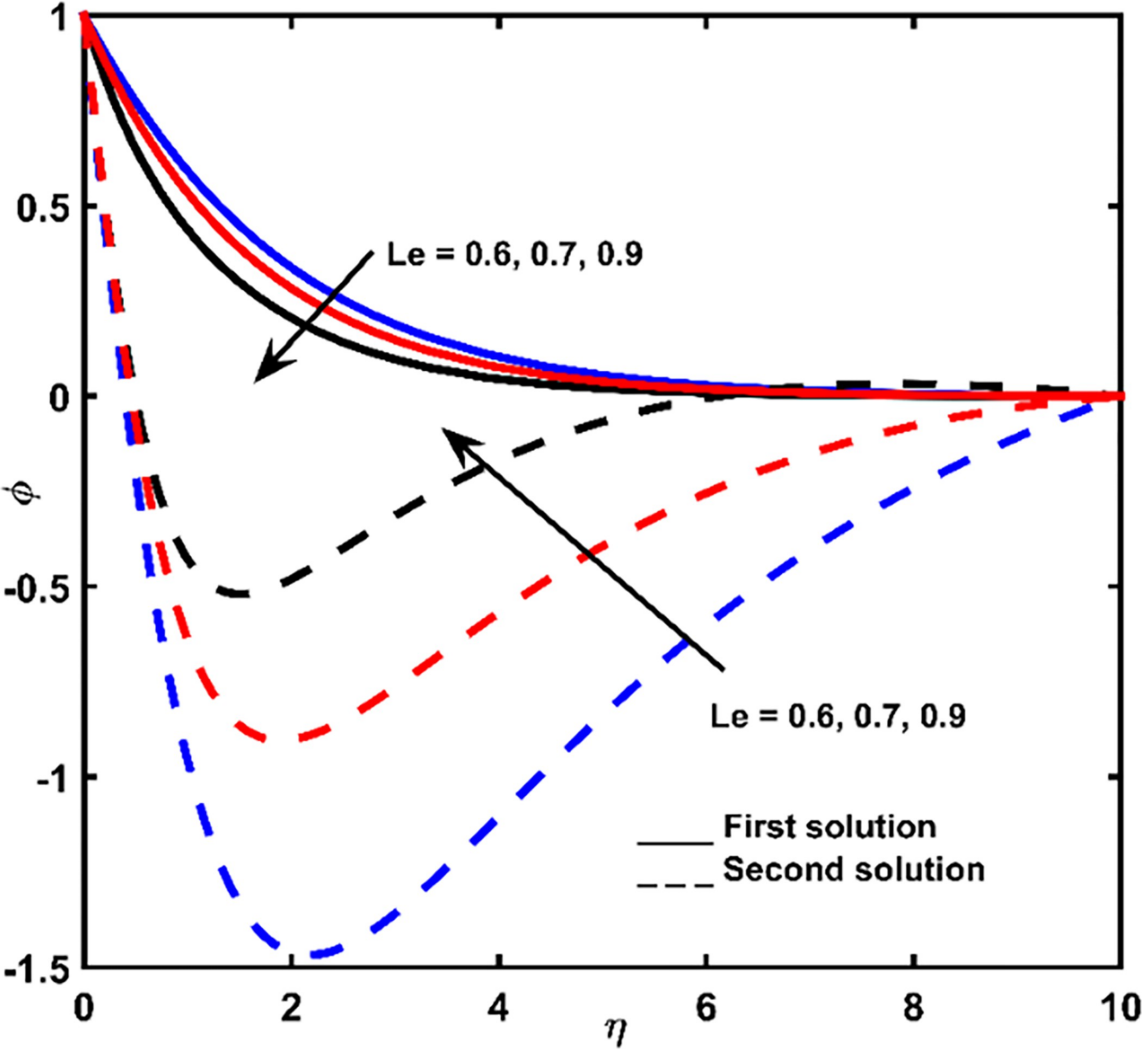
Concentration impact regarding *Le* values.

**Fig 9:**
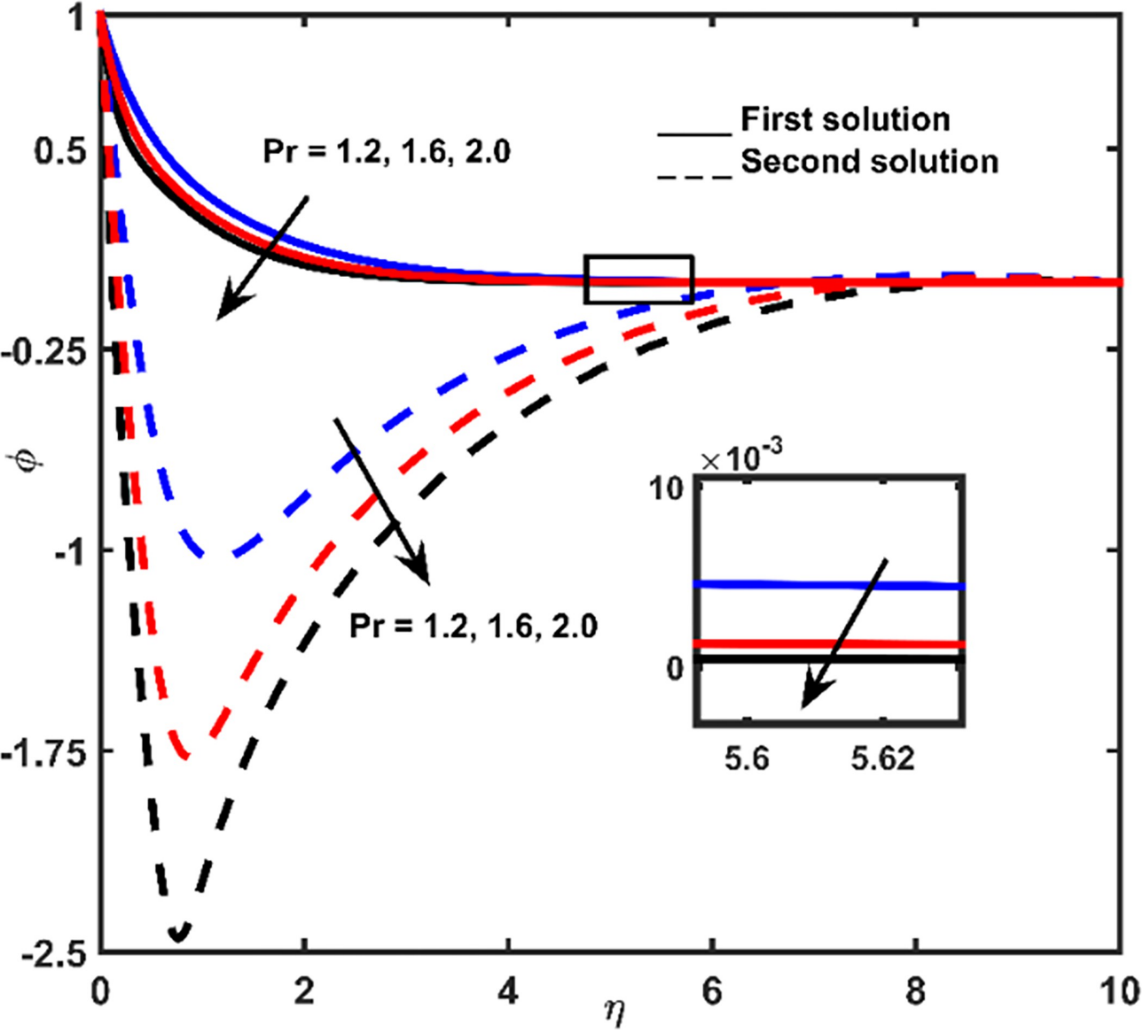
Concentration outline for diverse amounts of *Pr*.

Figs 7–9 display the influence of *Nb*, *Le*, and *Pr* on concentration profiles, respectively. With increasing values of *η*, a continuous deceleration in fluid concentration is observed until it tends to zero. According to the graph, when the distance from the shrinking surface is increased, the concentration rate becomes constant. Meanwhile, in a short distance of the surface, the lowest point of fluid concentration at a second solution is recorded. Hence, a rise in concentration is obtained and zero values are far from the shrinking surface (for larger *η*).

The rate of concentration in the first outcome becomes lessens due to the addition of *Nb*, *Le*, and *Pr* (Figs 7–9). For an improper fluid of definite momentum, higher *Le* effects low *Nb* coefficient, which must indicate in a lower concentration (Fig 8). The effect of *Pr* is to reduce concentration boundary layer thickness (Fig 9).

The concentration values for the second are ascended due to the improvement of the parameters *Nb* and *Le* (Figs 7 and 8) and descended with the addition of *Pr* (Fig 9).

### 3.6 Skin friction coefficient (SFC)

**Fig 10:**
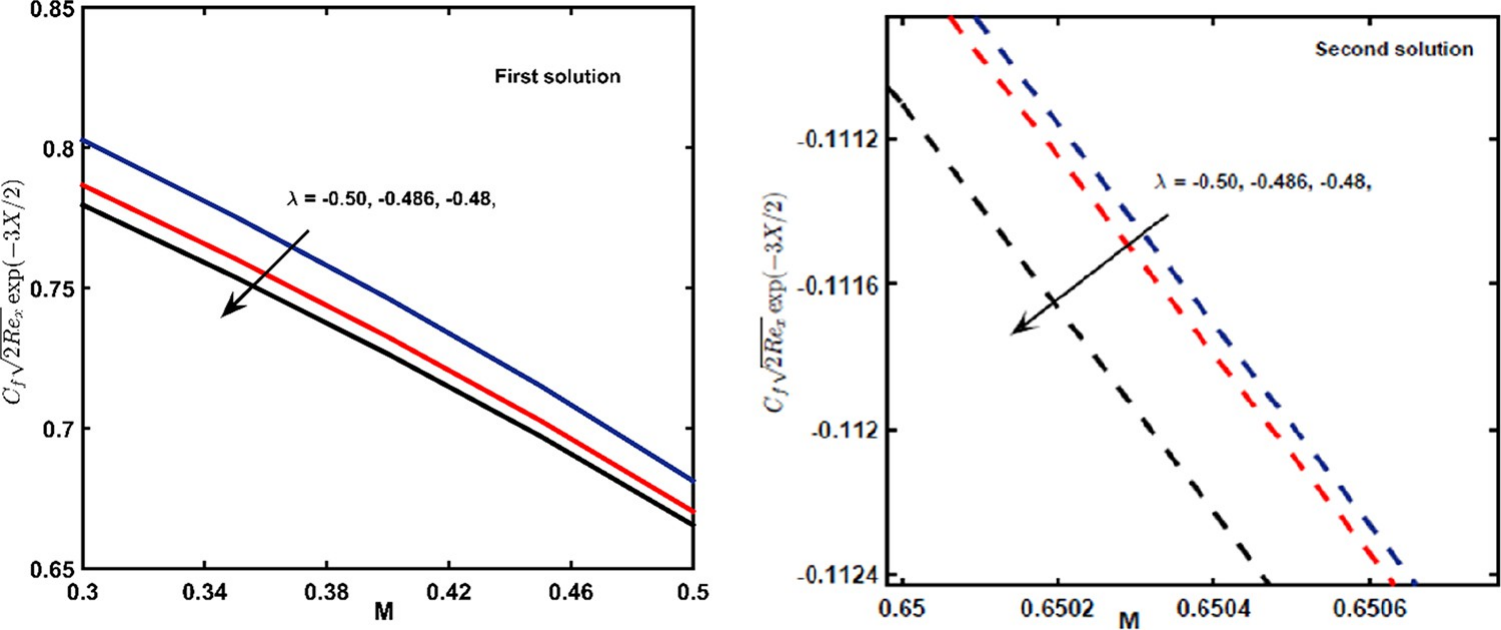
The graph of SFC with *M* of lessening parameter *λ*.

Change in SFC is depicted in Fig 10, which is according to *M* and *λ*. When a shrinking factor is enhanced, the rate of SFC increased for both solutions. Moreover, the effect of magnetic field *M* vanishes SFC values during the first solution.

### 3.7 Local nusselt number

**Fig 11:**
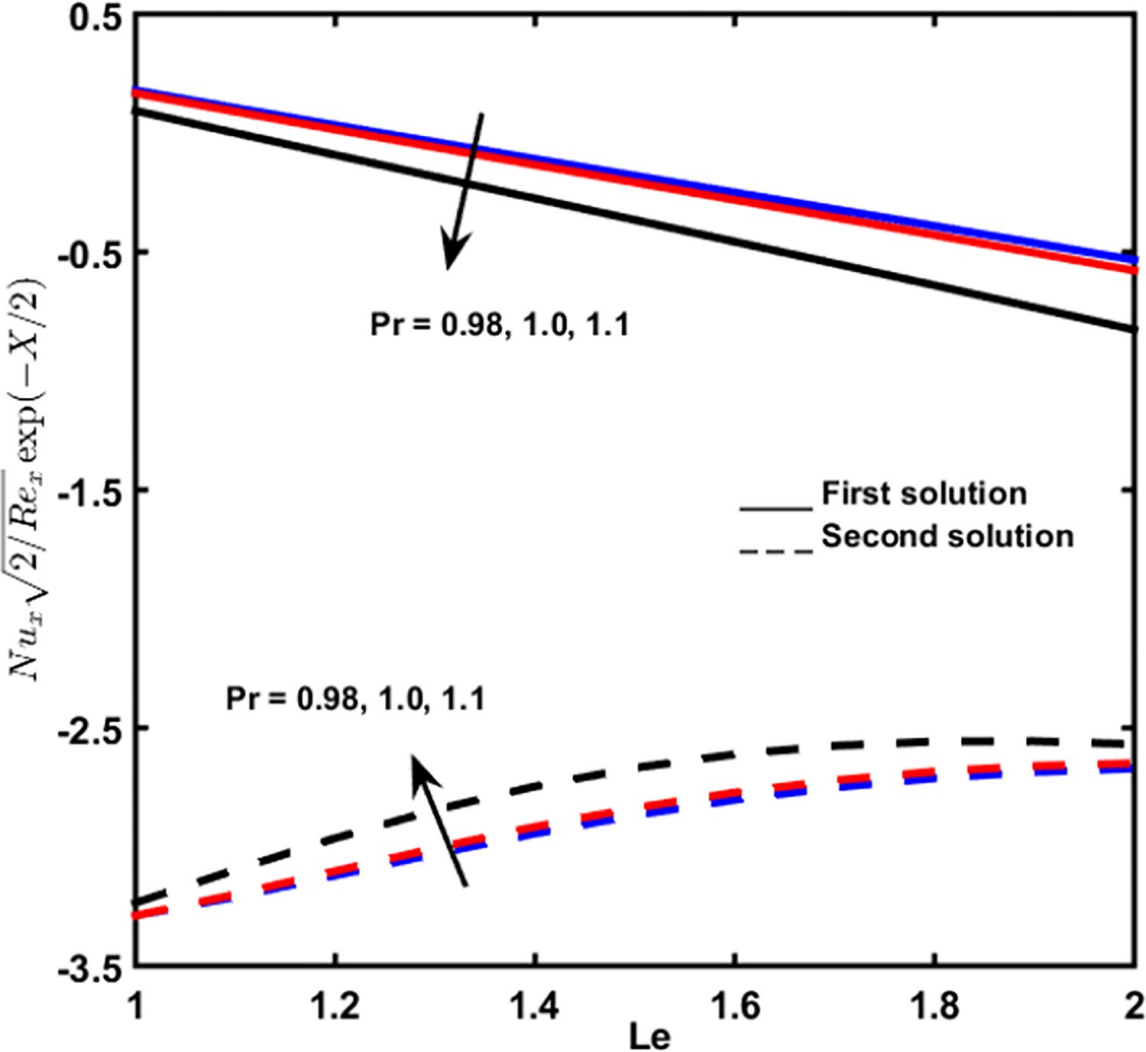
Nusselt number with Lewis number *Le* for diverse amounts of *Pr*.

Fig 11 displays the effect of *Pr* and *Le* on the variation of local Nusselt number. This variation is reduced due to the additional rate of *Pr* and *Le*. However, the local Nusselt number increases as *Pr* and *Le* increase.

### 3.8 Local sherwood number

**Fig 12:**
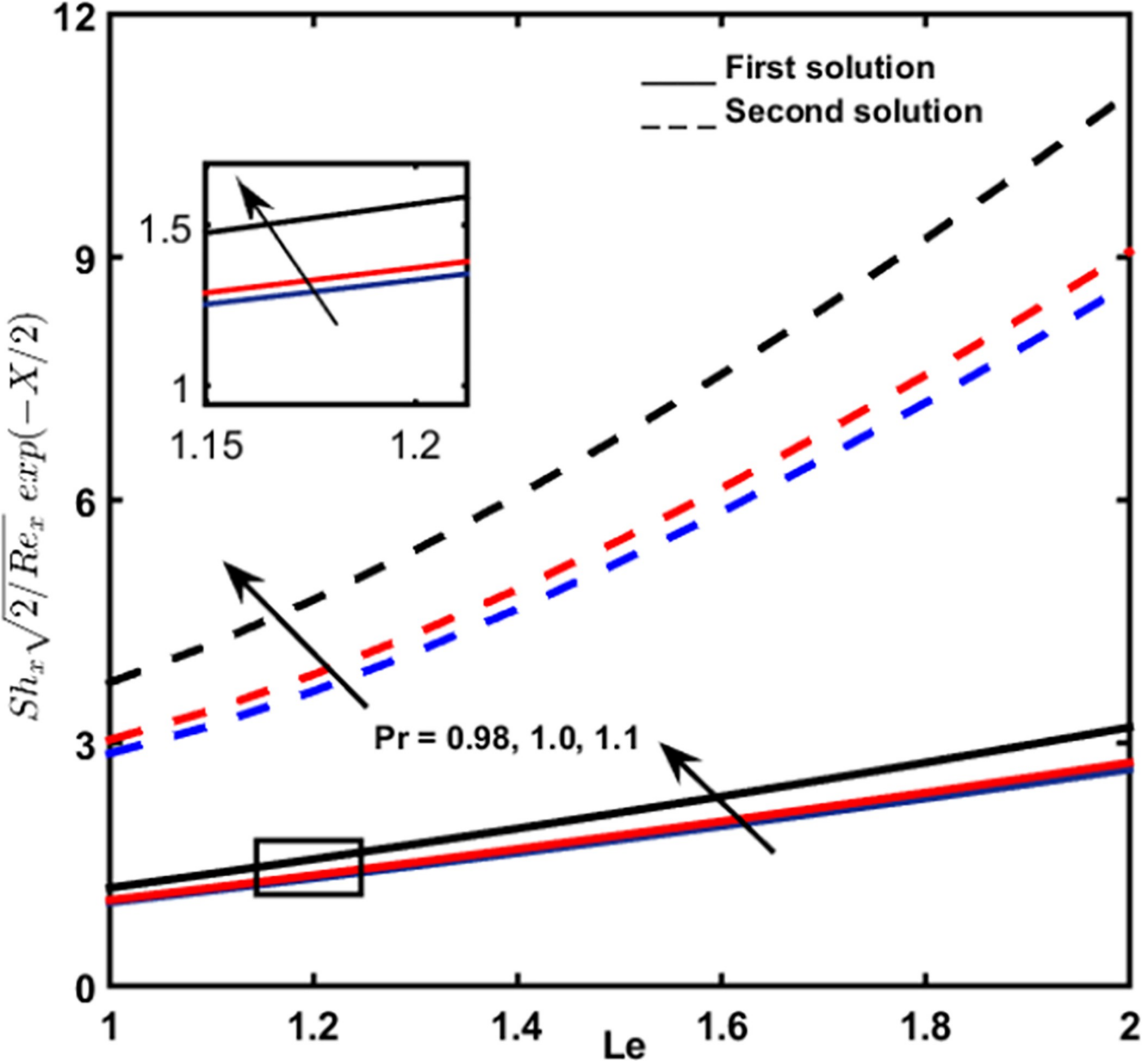
The local sherwood amount for *Le* for diverse amounts of *Pr*.

Fig 12 shows the effect of controlling parameter Lewis number on local Sherwood number, together with the different values of *Pr*. This graph displays that the implication of Lewis, as well as Prandtl number, contributes to the enhancement of the local Sherwood number.

## 4. Conclusion

The main objectives of this study are as follows: a) To describe the effect of the regulatory parameters on the model by developing the stable part in bvp4c Matlab program, to solve the final ODEs, b) To select the most stable and reliable numerical solution which occurs in the actual fluid state, by developing the stability analysis part. From the Results and Discussion section, it is proved that these objectives are successfully achieved in this study. Features of Maxwell-nanofluid mixed convective flow with Soret-Dufour impacts on the input material are reported. The liquid model is bound by a sloping sheet that is inclined. Numerical effects are modeled by the following factors: buoyancy-ratio, absorption, thermophoresis, magnetic force, Brownian motion, Lewis as well as Prandtl number. As the results in table and chart layout, the main conclusions of a reliable mathematical solution (first solution) are defined as below:

- An increase in speed magnitude is provided by the magnetic field along with the buoyancy ratio.
- Effect of Brownian diffusion *Nb*, Prandtl number *Pr*, and Lewis number *Le* are to enhance temperature values at all the range of fluid thickness (due to *Nb*) and the thinner fluid thickness (under the effect of *Le* and *Pr*).
- The decrement of concentration profile is caused by *Nb*, *Le*, and *Pr*.
- SFC is reduced when magnetic *M* is increased and the rate of lessening rate *λ* is decreased.
- The decrement of local Nusselt number is affected by *Le* and *Pr*, whereas the same parameters enhance local Sherwood number.
